# IHOG: Interval-Optimized Hamming-Weight-Oriented Grouping for Enhanced Side-Channel Leakage Detection

**DOI:** 10.3390/e28060662

**Published:** 2026-06-10

**Authors:** Jifang Jin, Tianqi Zhou, Ding Ding, Ye Huang, Bingqi Xie, Xiaoyi Duan

**Affiliations:** Beijing Electronic Science and Technology Institute, Beijing 100070, China

**Keywords:** Welch’s *t*-test, leakage detection, AES, power analysis attacks

## Abstract

The purpose of side-channel leakage detection is to determine whether or not there is side-channel leakage in the target cryptographic chip. The application of grouping (i.e., dividing the collected power traces into groups based on a property of the intermediate value, such as the Hamming weight of a byte or the bit value of an S-box output) in side-channel leakage detection is a research hotspot. The bit-level grouping mode and the byte-value grouping mode are proposed by previous scholars. However, the bit-level grouping mode does not match the byte operation architecture of cryptographic chips, resulting in an overly fine detection granularity and a high computational complexity. Although the byte-value grouping mode takes into account the byte operation architecture of cryptographic chips, it will cause unequal sizes of traces contained in two groups, reducing the test efficiency. In light of this, we propose the Interval-Optimized Hamming-Weight-Oriented Grouping (IHOG) Mode. IHOG groups data according to the Hamming weight (HW) of byte, dividing them into two groups with Hamming weights of {0, 1, 2, 3} and {5, 6, 7, 8}. In this way, it solves the problem of overly fine detection granularity and high computational complexity caused by bit-level grouping, and it also addresses the issue of unequal sample sizes and low test efficiency caused by the byte-value grouping mode. This paper verifies the effectiveness of the proposed IHOG method using four datasets, namely DPA v4, AES HD, Custom Dataset 1, and Custom Dataset 2. The results show that, compared with three existing grouping schemes such as HW value, bit value, and byte value, the IHOG scheme proposed in this paper increases the accuracy of leakage detection by 37.2%, 18.5%, and 146.3% respectively at the selected leakage points.

## 1. Introduction

Power information, running time and electromagnetic radiation will be leaked during the operation of a cryptographic chip, which may leak information about the intermediate state of the cryptographic chip [1]. Side-channel attack (SCA) can recover the key used by the target cryptographic chip by analyzing its leakage information. Typical SCA includes power analysis attacks [2], timing attacks [3], and so on.

As an important style of SCA, power analysis attack has been developed to be the most popular one. In 1999, Kocher proposed the Differential Power Analysis (DPA) [4] and used it to successfully recover the key used by a DES implementation. In addition to DPA, commonly used power analysis attacks include Simple Power Analysis (SPA) [2], Correlation Power Analysis (CPA) [5], Template Attack (TA) [6], Collision Attack (CA) [7] and deep learning-based side-channel analysis (DLSCA) [8,9], and high-fidelity model extraction attacks via remote power monitors [10]. Recent studies on AI-assisted assessment [11] further demonstrate the potential of machine learning in streamlining leakage detection [8,12,13,14]. Multilabel deep learning approaches have shown efficiency in side-channel attacks, further validating the role of AI in leakage analysis [15,16]. The advantage of the power analysis attack is that it only needs to collect the power consumption of the target cryptographic chip, and it does not need to do physical damage to the chip like the invasive attack [17]. The high efficiency of the attack and its undetectable characteristic poses a serious threat on the security of different types of cryptographic chips, including those based on algorithms like ECC where hybrid countermeasures have been developed to mitigate side-channel and fault attacks [18].

In practice, it is of great significance to detect whether or not there is any leakage related to the processing of sensitive data and to evaluate the resistance of a cryptographic chip to the power analysis attack. The ISO/IEC 17825 [19] standard then delineates the requirements and the detection techniques in order to evaluate the side-channel resistance of a cryptographic chip.

Leakage detection techniques are designed to identify possible leakage points contained in the measured traces related to the processing of sensitive data, and leakage assessment can be performed by a third-party assessment laboratory that evaluates the security of a cryptographic chip after chip production is complete [20]. It helps designers to have a preliminary knowledge of the security level of cryptographic chips. In this way, the chip designers can enhance the security of cryptographic chips by adding countermeasures to make sure that cryptographic chips are secure in application.

In addition, leakage detection techniques can be used to divide samples contained in the measured power traces into two groups according to a certain threshold, i.e., leakage points and non-leakage points [21]. The leakage points can be considered as the attack vulnerability point, and they can also be selected as the feature points [1] which contain information about the sensitive data. Performing power analysis attacks on leakage points can effectively reduce the time consumption and significantly optimize the efficiency.

Although side-channel attacks are effective against various cryptographic implementations (software, FPGA, cloud, etc.), this paper focuses on hardware chips due to their widespread use in security-critical applications.

### 1.1. Our Contribution

It is obvious that the choice of test positions as well as the grouping mode is crucial to achieve an accurate side-channel leakage detection. In light of this, the Interval-Optimized Hamming-Weight-Oriented Grouping (IHOG) leakage detection mode is proposed. In addition, the leakages at other positions are detected. In detail, the contribution of this paper is as follows.

(1) Research on the grouping mode: IHOG mode is proposed to perform leakage detection for cryptographic chips that follow the byte operation architecture. This grouping mode divides power traces into two groups according to whether the byte HW is in the interval of {1, 2, 3, 4} or the byte Hamming weight (HW) is in the interval of {5, 6, 7, 8}. In this way, it not only solves the problem that bit-level grouping leads to overly fine detection granularity and induces higher computational complexities, but also addresses the issue that byte-value-based grouping causes unequal sizes of traces in two groups and reduces the test efficiency.

(2) Research on test positions: It is not advisable to solely use the output of the S-box during the operation of the AES cryptographic chip as the test position. Instead, other operations such as the S-box input and MixColumns output should also be considered as test positions. In this paper, experiments have been carried out to verify that there are indeed leakage points at these positions.

(3) Evaluate the generalizability of IHOG: The proposed scheme has been extensively evaluated on two 8-bit MCUs (ATMEL AVR-163 and MEGA-128), one 32-bit MCU (STM32F103C8), and an FPGA (Xilinx Virtex-5) using four distinct datasets. The experimental results demonstrate that our approach consistently outperforms existing grouping schemes.

### 1.2. Structure of This Paper

Section 1 focuses on the background of the study and the current status of the research. Section 2 describes the process of the AES-256 algorithm and the principles of Welch’s *t*-test. In Section 3, the grouping modes as well as the selection of the test locations are discussed, and finally the necessity of repeating the test is explained. Section 4 presents the experimental results and useful conclusions can be obtained. Section 5 summarizes the whole paper and shows the future research directions.

## 2. Related Work

Research on the leakage detection of cryptographic chips can be traced back to the pioneering work proposed by Goodwill et al. [22] in 2011, which for the first time introduced Welch’s *t*-test into the leakage detection of the AES cryptographic chip. In 2012, the test vector leakage assessment (TVLA) is proposed by Cooper et al. for side-channel leakage detection [23]. After that, many scholars have conducted relevant research in these aspects, mainly focusing on the following aspects.

### 2.1. Research on the Detection Algorithm

Statistical hypothesis testing tools can significantly influence the accuracy of leakage detection. The traditional *t*-test remains a classical detection tool [23]. In 2016, Schneider et al. systematically investigated its implementation framework across diverse application scenarios, validating its universality in fundamental leakage assessment [24]. Ding et al. proposed a leakage detection scheme based on paired *t*-tests, aiming to improve both the accuracy and reliability of leakage detection [25]. However, the accuracy of conventional moment estimation approaches are influenced by the problem of false negatives when handling non-Gaussian distributions or small-sample data. In order to accurately perform leakage detection in these cases, Schneider et al. (2015) innovatively introduced the chi-square test to construct a leakage assessment model, effectively mitigating false negative rates through distributional morphology analysis [26]. Bronchain proposes a leakage detection method based on Hotelling’s $T^{2}$ test, which significantly enhances the detection sensitivity and accuracy when leakage signals are distributed across multiple sampling points in the power traces [27]. Zhou et al. proposed a leakage detection method based on the Kolmogorov–Smirnov (KS) test [28]. The results demonstrate that the KS test exhibits significantly better robustness compared to the traditional TVLA approach [29].

### 2.2. Solve the False Negative Problem in Detection

Balasch et al. investigated the trade-off between the choice of statistical threshold and the rates of false positives and false negatives. Their findings indicate that higher thresholds tend to increase the likelihood of false negatives, whereas lower thresholds are more prone to result in an increase in false positives [30]. Recent advancements have introduced information-theoretic perspectives to quantify information leakage and determine lower/upper bounds, offering a theoretical foundation for leakage assessment [31]. In 2018, Moradi et al. proposed a leakage detection method that combines the chi-squared test with TVLA, effectively mitigating the issue of false-negative errors caused by an insufficient number of data bins [32]. In 2021, Wang et al. [33] proposed the Bartlett-F test methodology, integrating Bartlett’s test with multi-class F-testing for leakage detection. This approach demonstrates enhanced sensitivity in scenarios where the difference in means between power traces contained in different groups are small. In 2023, the two-sample Kolmogorov–Smirnov test was strategically applied to z-value sequences derived from non-specific TVLA under varied sample sizes. This implementation reduces the probability of false negatives in TVLA when dealing with a limited number of power traces [34]. In 2024, the maximum mean discrepancy (MMD) technique was introduced [35,36], which extracts multivariate joint features from power traces, effectively circumventing the limitations of single-sample-point detection techniques.

### 2.3. Research on Grouping

The optimization of the sample grouping strategy has a decisive influence on the detection sensitivity. In [37,38], it is proposed to divide the data into two groups based on whether the byte HW is a certain value and use byte values for grouping. In [22], the bit values of the S-box output are used for grouping, and each bit value is tested separately. Those with a bit value of 0 are grouped together, and those with a bit value of 1 are grouped together. Those with a certain byte value are grouped together, and those with other byte values are in another group.

### 2.4. Limitations of Existing Techniques

Although many scholars have conducted research in this area in the early stage and proposed some detection techniques, there are still significant bottlenecks in the implementation process: (1) The bit-level grouping mode proposed by previous scholars does not match the byte operation architecture of cryptographic chips, resulting in an overly fine detection granularity and a high computational complexity; (2) Although the byte-value grouping mode takes the byte operation architecture into account, it will cause the sample sizes of the two groups to be unequal, reducing the test efficiency [39]; (3) There is a lack of theoretical guidance for the selection of test positions. Most of the existing research is limited to the output of the S-box; (4) The sizes of the samples in two groups will be unequal and the variances of the samples in two groups will also be unequal. Therefore, it is necessary to propose a leakage detection algorithm that performs well in this situation.

## 3. Preliminaries

### 3.1. AES

**Figure 1:**
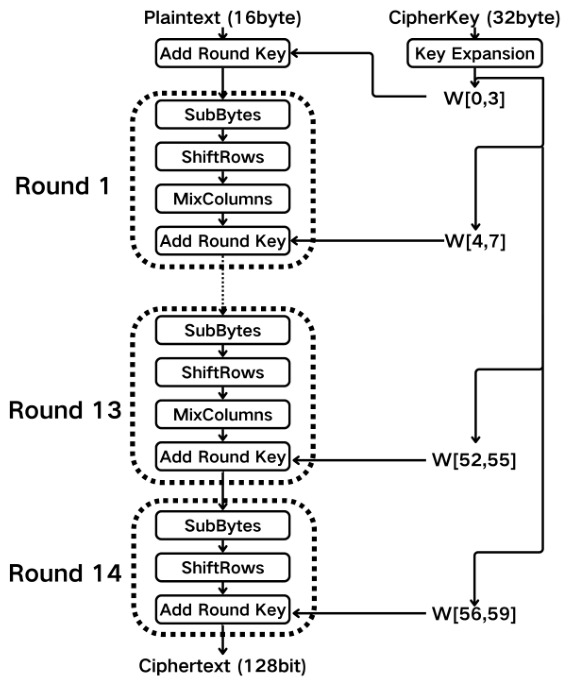
AES-256 algorithm encryption process.

The Advanced Encryption Standard (AES) stands [38] as one of the most widely used symmetric encryption algorithms. As is shown in Figure 1, AES-256 encryption is composed of 14 rounds of transformation; each contains four computation components. In the AES algorithm, the SubBytes operation is a non-linear transformation, which can play a role of confusion and diffusion of data in the encryption process. SubBytes relies on the non-linear component S-box, whose non-linear properties are reflected in the fact that when the input data is different in one single bit, its output will be different in multiple bits. Its good non-linear properties can effectively resist traditional cryptanalysis techniques. However, the non-linear property of the S-box makes it exploitable by side-channel analyzers to implement power analysis attacks.

### 3.2. Welch’s t-Test

The *t*-test is a hypothesis test in statistics that measures the difference between two means of subsets with unequal variances but the same sample size. Welch’s *t*-test is more reliable in cases where the sample sizes of the two subsets are not equal. There are three steps involved in assessing the leakage contained in the measured traces.

Step 1: Set the null hypothesis. The null hypothesis is set as there is no power leakage in the measured traces, and the power traces are divided into two groups according to a division strategy and do not differ statistically with high confidence.

Step 2: Divide the measured traces into two groups. For the specific *t*-test, a certain intermediate value during the operation of AES is chosen as the test position, and n power traces are divided into two subsets ($L_{0}$ and $L_{1}$) according to two possibilities of this value selection, i.e., $L_{0}=\left\{T_{i}|m_{i}=0\right\}$, $L_{1}=\left\{T_{i}|m_{i}=1\right\}$. When the intermediate value is a single bit, there are two possibilities for the value, i.e., 0 and 1. However, when the intermediate value is not a single bit but a multi-bit value, it can be grouped according to whether the value is α or not. $L_{0}$ and $L_{1}$ can be expressed as $L_{0}=\left\{T_{i}|m_{i}=\alpha \right\}$, $L_{1}=\left\{T_{i}|m_{i}\neq \alpha \right\}$.

Step 3: Calculate the sample sizes, means, and variances of $L_{0}$ and $L_{1}$, which can be expressed as $\left(n_{0},u_{0},S_{0}^{2}\right)$ and $\left(n_{1},u_{1},S_{1}^{2}\right)$. Then, the t statistic can be calculated in Equation (1).

(1) $$\begin{array}{c} t=\frac{u_{0}-u_{1}}{\sqrt{\frac{S_{0}^{2}}{n_{0}}+\frac{S_{1}^{2}}{n_{1}}}} \end{array}$$

The degrees of freedom can be calculated in (2).

(2) $$\begin{array}{c} v=\frac{{\left(\frac{S_{0}^{2}}{n_{0}}+\frac{S_{1}^{2}}{n_{1}}\right)}^{2}}{\frac{{\left(\frac{S_{0}^{2}}{n_{0}}\right)}^{2}}{n_{0}-1}+\frac{{\left(\frac{S_{1}^{2}}{n_{1}}\right)}^{2}}{n_{1}-1}} \end{array}$$

The probability density function of the t-distribution can be computed according to the degree of freedom, and its computation process is shown in (3), which defines the standard t-distribution’s probability density function in statistics. In (3), Γ() denotes the Gamma function, v denotes the degrees of freedom parameter, while t is the test statistic.

(3) $$\begin{array}{c} f\left(t,v\right)=\frac{\Gamma (\frac{v+1}{2})}{\sqrt{\pi v}\Gamma \left(\frac{v}{2}\right)}{\left(1+\frac{t^{2}}{v}\right)}^{-\frac{v+1}{2}} \end{array}$$

Then, the probability that the null hypothesis holds can be computed in (4).

(4) $$\begin{array}{c} p=\int_{\left|t\right|}^{\infty }f\left(t,v\right)dt=2F\left(-|t|,v\right) \end{array}$$

where F is the distribution function, whose calculation can be shown in (5).

(5) $$\begin{array}{c} F\left(t,v\right)=\frac{1}{2}+t\Gamma \left(\frac{v+1}{2}\right)\frac{2^{F_{1}\left(\frac{1}{2},\frac{v+1}{2},\frac{3}{2},-\frac{x^{2}}{v}\right)}}{\sqrt{\pi v}\Gamma \left(\frac{v}{2}\right)} \end{array}$$

Note that in (5), $2^{F_{1}}$ is a hypergeometric function.

When the *p*-value calculated with (4) at a sampling point is less than the threshold (this value is usually set to 4.5), the variance of the power consumption at that sample point can be large with a high confidence, and it can be determined as a leakage point. Therefore, the setting of the threshold value is very important in order to determine the leakage point. Because the computational complexity of the complete *t*-test process can be high, in the actual leakage detection a simplified statistic p can be computed in (6). One can see that in the computation of the simplified statistic p, the values of f and F are not considered. When the sample size is more than 1000, setting the threshold for acceptance or rejection of the null hypothesis to 4.5 will result in a *t*-test with an accuracy of more than 99.999% [1].

(6) $$\begin{array}{c} p=2F\left(t=\pm 4.5,v>1000\right)<10^{-5} \end{array}$$

### 3.3. HW Model

HW denotes the number of non-zero elements in a string [40]. The HW model is a leakage model commonly used in power analysis attacks. The power consumption and the electromagnetic leakage captured by the attacker have a linear relationship with the HW of the processed intermediate data, based on which differential side-channel analysis can be implemented [41]. In light of this, this paper introduces the HW model into the *t*-test grouping.

## 4. IHOG-Based Leakage Detection with Welch’s *t*-Test

### 4.1. HW Characteristics of the S-Box Output of AES

The values of the S-box output of AES are within the set [0, 255]. Therefore, there are nine possible values for the HW of the S-box output of AES. The HW of the S-box output of AES can approximately follow the normal distribution, and the distribution of the values of the HW of the S-box output of AES is shown in Table 1.

In TA or DLSCA, two types of models can be constructed for S-box outputs: the full-byte model and the HW model. Compared with the full-byte model, the HW model exhibits higher classification accuracy due to its lower dimensionality of classification and linear correlation with the leakage power consumption of circuits, thus being widely adopted in practice [42]. Recent work on DL-SCA, such as the 3D power surface approach for input recovery [43,44], and the comprehensive survey on machine learning in side-channel attacks [45], further demonstrates the potential of deep learning in side-channel analysis. Furthermore, emerging threats to AI systems (e.g., adversarial attacks, model extraction, and side-channel attacks) highlight the need for robust leakage detection frameworks in cryptographic implementations [46].

Cryptographic chips typically operate in byte units; therefore, leakage detection must fully incorporate their 8-bit byte operational characteristics, employing byte-level grouping and verification strategies. This paper extends the HW model, which is widely used in side-channel analysis, to the task of energy information leakage detection, enabling leakage detection through byte-level grouping.

### 4.2. IHOG: Interval-Optimized Hamming-Weight-Oriented Grouping

In order to address the issues of high computational complexity caused by the mismatch between the bit-level grouping mode and the byte operation architecture of cryptographic chips, as well as the decreased efficiency caused by the byte-value grouping mode, this paper proposes IHOG that takes into account the byte operation architecture of cryptographic chips, as is shown in Table 2.

**Figure 2:**
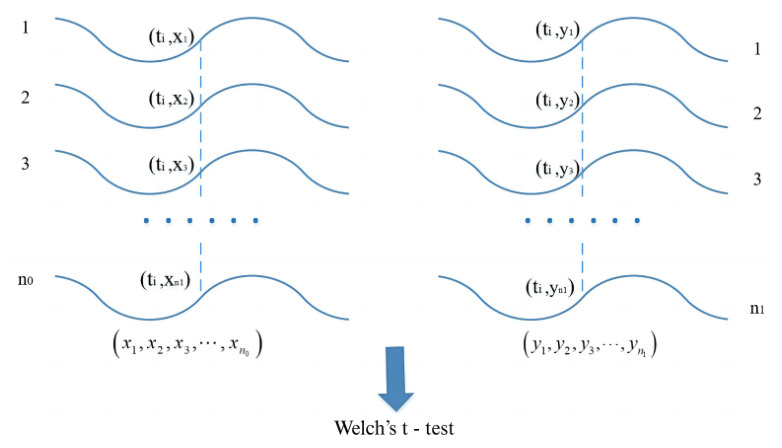
Schematic diagram of Welch’s *t*-test.

Mode I, the IHOG mode, divided the power traces into two groups, namely Subset I and Subset II. Subset I contains power traces related to the processing of the target intermediate value whose HW is in the interval HW = {0, 1, 2, 3}, and Subset II contains power traces related to the processing of the target intermediate value whose HW is in the interval HW = {5, 6, 7, 8}. Then, a Welch’s *t*-test is conducted on the two groups. The schematic of Welch’s *t*-test is shown in Figure 2.

Mode II, which is based on [35], can divide power traces into two distinct groups according to the intervals of the HW of the S-box output of AES. The difference between mode I and mode II is that mode I uses the values of the HW of the S-box output of AES as the basis for classification, and power traces contained in the dataset are divided into two groups according to the intervals of the values of the HW of the S-box output of AES. Comparatively, mode II divides power traces contained in the dataset into two groups according to the values of the HW of the S-box output of AES. In light of this, the difference between the sizes of traces divided into two groups under mode I may be smaller than that under mode II.

Mode III (bit value) is proposed in [1]; in this mode each bit value is tested separately, and power traces contained in the dataset related to a bit value of 0 are grouped together, while power traces related to a bit value of 1 are grouped together. Then, power traces contained in two groups are used for leakage detection. Totally, leakage detection should be performed 128 times to detect the leakage related to the processing of each bit of 16 S-box outputs of AES.

Mode IV (byte value) is proposed in [35]; in this mode each byte is regarded as a unit. Note that there are $2^{8}$ = 256 possible values for each byte, and power traces contained in the dataset are divided into two groups according to a possible value of each byte. Therefore, Model IV needs to perform leakage detection 512 times for each byte, which means that the amount of time consumption of mode IV can be large.

The aim of this paper is to find the grouping mode that can obtain better detection results and to study the effect of different test positions on detection results. Considering that the output of the first-round S-box is commonly chosen as the target position in power analysis attacks, when studying the optimal grouping mode, each of 16 S-box outputs of the first-round AES encryption can be chosen as the target intermediate value.

**Figure 3:**
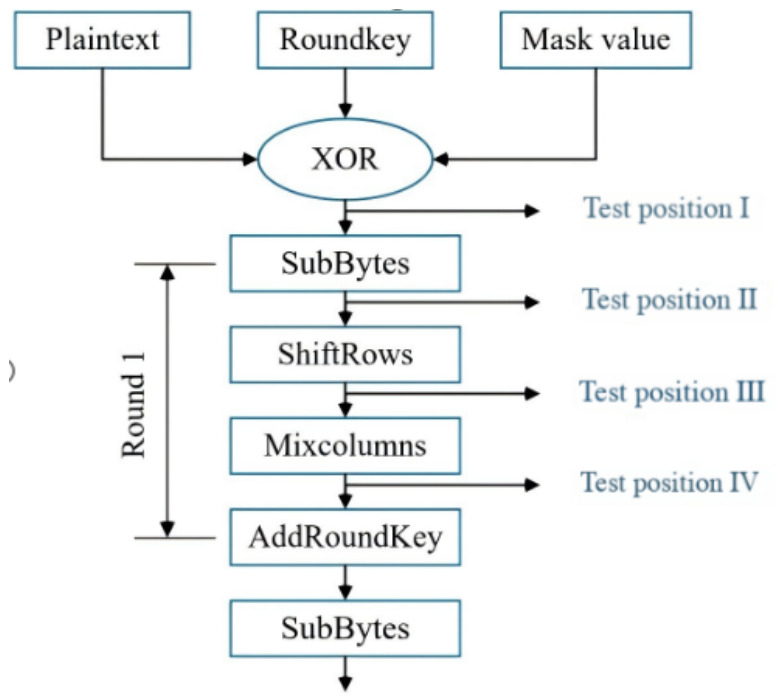
Schematic representation of the four test positions of Welch’s *t*-test.

The four grouping modes shown in Table 2 are used to divide power traces contained in the dataset into two groups, and then Welch’s *t*-test is carried out. A schematic diagram of Welch’s *t*-test after grouping is shown in Figure 3. The test is performed on each sample of the measured power traces. For the IHOG mode, there are eight possible configurations due to the eight possible choices of the intervals. Similarly, the bit-level grouping mode induces eight distinct configurations per byte. In contrast, the byte-value grouping mode requires 256 configurations per byte, while the HW-value grouping mode induces five configurations. In this paper, the best grouping mode and the effect of different test positions on the test results is known by performing experimental evaluations.

### 4.3. t-Test Test Positions

In addition to the grouping mode, the selection of the appropriate intermediate value, i.e., the test position, is crucial for the implementation of Welch’s *t*-test. The focus of this paper is on the AES-256 cryptographic chip. The S-box operation is the non-linear transformation in the AES-256 algorit HW, and the non-linear property makes the S-box operation a good option for power analysis attacks. The first-round S-box output is usually used in power analysis attacks as the target location. In [1], Goodwill et al. set the S-box output of a middle round operation of the AES cryptographic chip as the test position, and the specific value of each bit of the S-box output is used as the basis for grouping. Theoretically, the side-channel leakages related to the processing of other intermediate states also exist. Consequently, research on the leakage detection of other intermediate transformations of a block cipher is needed to form a more complete mechanism for leakage detection of block cipher-based cryptographic chips. In light of this, the four positions of the first-round encryption process are set to be the test positions to carry out the experiments respectively, and the test positions are shown in the schematic diagram of Figure 3. By comparing the distribution intervals of leakage points obtained from the experiments, the influence of different positions on the effectiveness of leakage detection is known. The number of repeated tests adopted in this paper is 512.

### 4.4. Repeated Tests

As is shown in (6), the accuracy of Welch’s *t*-test can reach more than 99.999% when the threshold is set to 4.5. In the experiment, however, each trace contained 400,000 samples. The very large number of samples makes it impossible to exclude the possibility of false positives at some sampling points. Performing a second, independent yet identical experiment to the first experiment as a repeat test is conducive to reducing false positives. For example, when 20,000 traces with the same fixed key provided by DPA contest v4 are selected, 10,000 of them can be used in Welch’s *t*-test, while the remaining 10,000 can be used to perform a repeated test. The two sets of samples are independent. For a sampling point on the power trace, it can only be determined as a leakage point if the *t*-statistic at that point is larger than the threshold in two independent experiments at the same time. This is because if the *t*-statistic exceeding the threshold at a particular time is a chance event, it is unlikely to be repeated in the next replication of the experiment [1].

### 4.5. Selection of the Testing Algorithm

The *t*-test contains Student’s *t*-test and Welch’s *t*-test; when using the IHOG mode proposed in Section 3.3 to perform leakage detection, an important question is which testing algorithm should be selected as the test tool.

Theoretically, since the plaintext during encryption is not completely uniform, there will be situations where the sizes of the power traces contained in two groups are unequal and the variances of the power traces contained in two groups are unequal. Welch’s *t*-test is very robust for the mean test in cases where the sizes of the two samples are unequal and the variances of the two sample values are unequal. Therefore, it is more appropriate to choose Welch’s *t*-test as the tool.

Since Welch’s *t*-test is robust for the mean test in cases where the sizes of the two samples are unequal and the variances of the two sample values are unequal, both theoretically and experimentally, it is proven that when using the *t*-test for leakage detection, Welch’s *t*-test, which is suitable for the situation where the variances of the two samples are unequal, should be selected instead of Student’s *t*-test.

## 5. Experiments and Analysis

To comprehensively verify the effectiveness and generalizability of the proposed grouping scheme, we conducted validation experiments using datasets collected from widely deployed 8-bit MCUs, 32-bit MCUs, and FPGA platforms. Considering that modern cryptographic algorithm implementations generally integrate protective mechanisms, the experimental design specifically covers different security levels: on 8-bit MCU platforms, we selected both unprotected and protected implementation samples respectively, supplemented by an unprotected implementation sample on FPGA and a 32-bit MCU platform, thus forming a validation framework consisting of 5 representative datasets. By systematically comparing the proposed scheme with the four grouping methods described in Section 3 on these datasets, we systematically validate the superiority of the proposed scheme.

### 5.1. Dataset

**DPA v4**: This dataset was acquired by the research team at Télécom Paris, Institut Polytechnique de Paris, in compliance with international standards [47]. The power traces were collected from an ATMEL AVR-163 (8-bit) microcontroller executing an AES-256 algorithm protected by the Rotating S-box Masking (RSM) countermeasure [48]. For our experiments, we selected 20,000 traces encrypted with the same 256-bit fixed key.

**AES_HD**: Provided by the AISyLab, this dataset captures power consumption of an unprotected AES implementation on a Xilinx Virtex-5 FPGA (SASEBO GII evaluation board) [49]. Our study utilizes 20,000 traces encrypted with a fixed key.

**Custom Dataset1(CDS1)**: We collected this dataset using a ChipWhisperer-Lite (CW1173) platform [50], monitoring power consumption during unprotected AES execution on an ATmega128 (MEGA-128) microcontroller. The analysis is based on 20,000 traces with a shared key.

**Custom Dataset2(CDS2)**: We collected this dataset using a CrackNuts platform [51], monitoring power consumption during unprotected AES execution on an Arm 32-bit Cortex-M3 CPU(STM32F103C8). The analysis is based on 10,000 traces with a shared key.

The prerequisite for the implementation of the *t*-test is that the samples are required to obey a normal or approximately normal distribution, and when the sample size is greater than 30, the data can be considered to be approximately normally distributed. In the experiment, the sample size of the trace set is greater than 30, which meets the prerequisite for the implementation of the *t*-test.

### 5.2. The Optimal Values of Various Grouping Modes

To identify the optimal grouping strategy among the several grouping models listed in Table 2, this study conducted verification experiments on the DPAV4 dataset to determine the optimal scheme for each grouping method. This provides a reliable basis for subsequent experimental design on other datasets.

#### 5.2.1. The Optimal Grouping Mode for Method II

**Figure 4:**
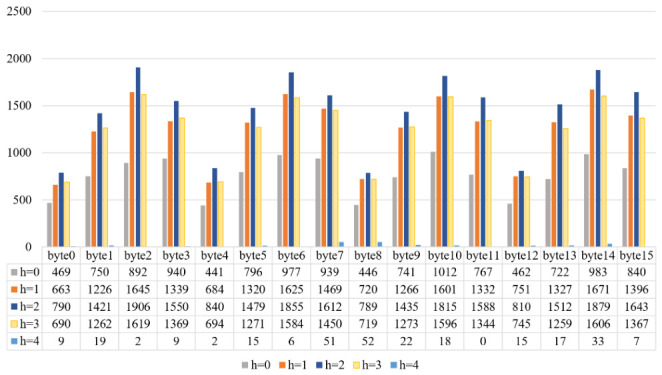
The leakage points obtained under Method II.

This grouping mode is applied to the AES algorithm and the leakage points are successfully detected. By comparing the numbers of leakage points obtained under different modes, it can be seen that grouping according to whether the Hamming weight value of each byte at the test position is 2 or not gives better detection results, as is shown in Figure 4.

#### 5.2.2. The Optimal Grouping Mode for Method III

**Figure 5:**
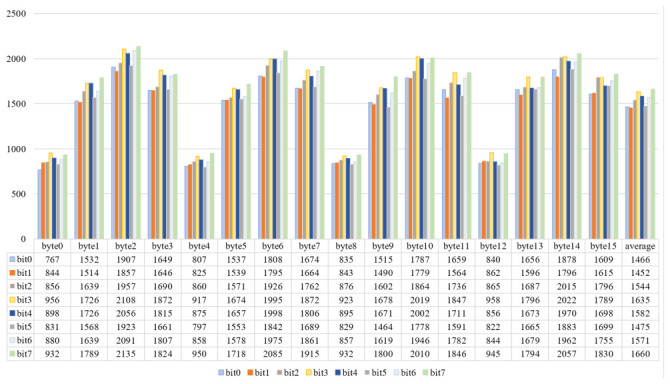
The leakage points obtained under Method III.

Under the bit-level grouping mode, one should perform leakage detection for each bit of the 16 S-box outputs of the first round of AES encryption. That is, one should divide the power traces according to whether or not each bit of the 16 S-box outputs is 0 or 1, and then use Welch’s *t*-test to obtain the *t*-test statistic graph. In light of this, 128 × 2 = 256 experiments should be performed to obtain leakage points related to the processing of each bit value, as is shown in Figure 5. It can be seen that the numbers of leakage points related to the processing of different bits of an S-box output can be about the same. According to statistics, a larger number of leakage points can be detected for the bit7 of different bytes.

#### 5.2.3. The Optimal Grouping Mode for Method IV

**Figure 6:**
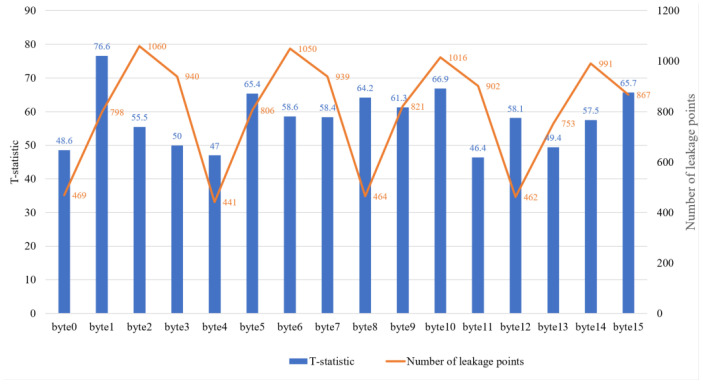
The leakage points obtained under Method IV.

Under the byte-value mode, power traces should be divided into two groups according to whether the value of the target byte is a certain value or not. In Figure 6, a *t*-statistic is computed for each target byte. One can see that the number of selected leakage points related to the processing of different target bytes can be different. In addition, a larger *t*-statistic value does not represent a larger number of selected leakage points.

### 5.3. Experiment Results

#### 5.3.1. Protected AES-256 on 8-Bit Microcontroller (DPA v4)

Leakage point detection was performed on the DPA V4 dataset according to the four grouping schemes proposed in Section 3, and the results are presented in Table 3. As can be seen from the table, all four detection schemes are capable of identifying leakage points. However, the scheme proposed in this paper detects the maximum number of leakage points, followed by the groupings {0, 1, 2, 3} and {4, 5, 6, 7, 8}. Owing to the more balanced distribution of data in the two subgroups of the proposed grouping scheme, it yields better detection performance.

#### 5.3.2. Unprotected AES-128 on FPGA (AES_HD)

Since AES_HD runs on the Xilinx Virtex-5 FPGA of the SASEBO GII evaluation board, its signal-to-noise ratio (SNR) is extremely low due to the extensive parallel operations inherent in the hardware implementation of AES. The results are presented in Table 4, from which it can be observed that compared with other software-implemented datasets, AES_HD contains significantly fewer leakage points. In fact, under certain grouping schemes, there are even no detectable leakage points. Nevertheless, the grouping scheme proposed in this paper still achieves the best performance.

#### 5.3.3. Unprotected AES-128 on 8-Bit Microcontroller (CDS1)

The user’s dataset was collected using the ChipWhisperer-Lite (CW1173) evaluation board. Since the AES cryptographic algorithm implemented here has no protective measures and the signals acquired by the CW1173 feature a relatively high signal-to-noise ratio (SNR), a large number of leakage points can be detected despite the energy traces of the dataset containing only 5000 points. Furthermore, for the unprotected AES software implementation on an 8-bit microcontroller, the proposed scheme in this paper also achieves the best performance, as shown in Table 5.

#### 5.3.4. Unprotected AES-128 on 8-Bit Microcontroller (CDS2)

The CDS2 is collected from the CrackNuts evaluation board, with the AES algorithm deployed on a 32-bit Arm Cortex-M3 processor. Compared to conventional 8-bit MCUs, this processor exhibits a significantly lower signal-to-noise ratio (SNR). This hardware advantage is directly reflected in the experimental results: as shown in the table, the IHOG scheme achieves superior performance in leakage point detection (Table 6).

#### 5.3.5. Detection Time

In addition, to achieve accurate detection results, leakage detection also requires minimizing the leakage detection time as much as possible. Therefore, it is necessary to analyze the detection time required for Welch’s *t*-test under each grouping mode. Since each grouping mode needs to be repeatedly tested with a second dataset (including the repeated test), this factor needs to be taken into account when calculating the time consumption. Then, according to the test results, the time consumption required for a single detection under different grouping modes can be around 900 s. However, the bit-level grouping mode needs to group power traces according to the value of each bit of the target byte. Therefore, it requires a total of 8 × 2 × 900 s. The byte-value grouping mode needs to perform the experiments 256 × 2 × 900 s according to 256 possible values of each target byte. The HW-value grouping mode only needs to divide power traces according to whether or not the specific Hamming weight value of the target byte is a certain value, which requires 5 × 2 × 900 s. Meanwhile, the HW-interval grouping mode proposed in this paper only needs 1 × 2 × 900 s to divide power traces according to whether or not the specific value of each byte is within a certain range. When the leakage points related to the processing of 16 bytes are detected, the advantage of detection time can be more obvious as in Table 7.

#### 5.3.6. Results of the Optimal Grouping Mode at Different Test Positions

The experimental results in Section 4.2 demonstrate that, for the leakage detection task of each byte, the proposed IHOG mode outperforms the other three comparative modes. Therefore, this grouping mode is identified as the optimal scheme when investigating the influence of different inspection positions on the results of Welch’s *t*-test. Specifically, key transformation nodes in the first-round encryption process of the AES algorithm—including S-box input, S-box output, row shift output, and column mix output—are all designated as inspection positions. After grouping the trace data using the IHOG mode, Welch’s *t*-test is performed to obtain the leakage characteristics of each position.

**Figure 7:**
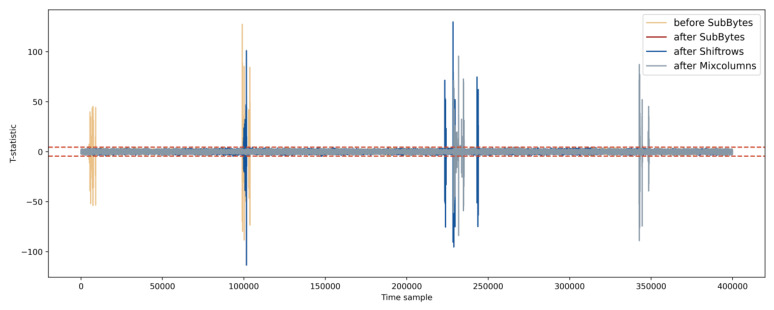
*t*-statistics obtained at different test positions (byte 0).

**Figure 8:**
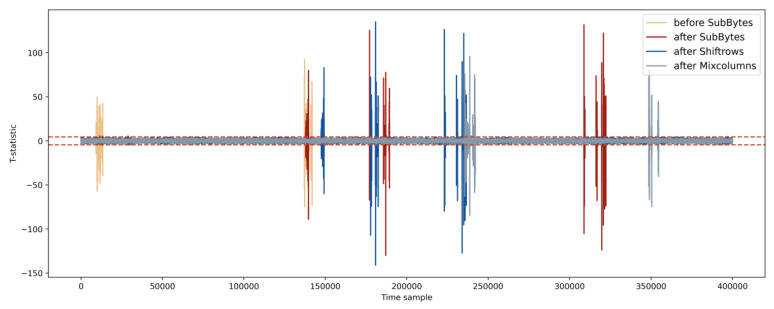
*t*-statistics obtained at different test positions (byte 1).

Figure 7 and Figure 8 show the detected leakage points at different test positions for byte 0 and byte 1. It can be noticed from Figure 7 that the leakage points related to the processing of the S-box output and the ShiftRows output are exactly the same shape. This phenomenon can be explained in conjunction with Figure 2. The four bytes of the first row do not move during the ShiftRows process. The same phenomenon can be found during leakage detection for byte 4, byte 8, and byte 12. In addition to the ShiftRows, the concentration areas of the leakage points obtained with the S-box input, S-box output, and MixColumns output as the test positions are not the same. Therefore, it can be known that if the leakage point detection is carried out only by taking the output of the S-box as the inspection position, not all leakage points can be obtained. When conducting leakage detection on the cryptographic chip, attention should be paid not only to the grouping scheme, but also to the influence of the inspection position on the inspection result. Therefore, performing experiments at different inspection positions can help evaluators to obtain the complete leakage area, so as to take targeted measures to enhance the protection ability of the algorithm. For example, not only the S-box output of the AES cryptographic chip should be protected, but also the other three operations of the AES cryptographic chip should be protected.

Qualitative analysis of leakage intensity at different test positions: As shown in Figure 7 and Figure 8, the S-box output achieves the highest *t*-statistic peaks and covers the broadest leakage ranges, followed by the ShiftRows output and MixColumns output, while the S-box input has the weakest leakage performance. In theory, the non-linear diffusion effect of the S-box brings higher information entropy and stronger power consumption correlation. The ShiftRows operation retains the side-channel leakage features generated by the S-box, whereas the MixColumns operation reduces the overall entropy value. Accordingly, we suggest prioritizing the S-box output for side-channel leakage detection. Meanwhile, ShiftRows and MixColumns positions can be adopted to implement comprehensive detection evaluation.

## 6. Conclusions

The purpose of side-channel leakage detection for cryptographic chips is to determine whether leakage exists, thereby preventing attackers from exploiting leakage points to steal sensitive information. To address the problems of high computational complexity, low detection efficiency, and the lack of systematic analysis of detection points in existing schemes, this paper proposes the IHOG (Interval-Optimized Hamming-Weight-Oriented Grouping) strategy. Fully adapted to the byte-operation architecture of cryptographic chips, IHOG groups power traces according to byte Hamming weight, dividing all traces into two categories with Hamming weight sets {0, 1, 2, 3} and {5, 6, 7, 8}. This method not only reduces the high computational overhead caused by the overly fine granularity of bit-level grouping, but also improves the low detection efficiency resulting from unbalanced sample sizes in byte-value grouping. In light of this, this paper proposes the IHOG mode. This method fully considers the byte operation architecture of the cryptographic chips. This grouping mode divides the traces according to the byte Hamming weight, grouping them into two sets with HW of {0, 1, 2, 3} and {5, 6, 7, 8} respectively. This approach not only solves the problem of excessive computational complexity caused by the overly fine detection granularity of bit-level grouping but also addresses the issue of reduced inspection efficiency due to unequal sample sizes in Byte-value grouping. Experiments conducted on the DPA v4, AES HD, Custom Dataset 1, and Custom Dataset 2 demonstrate that the proposed scheme outperforms bit-level-based and byte-value-based schemes in leakage point detection. Meanwhile, this paper also suggests that when detecting leakage points, not only the output of the S-box, but also other locations should be considered. Experiments have verified that there are also side-channel leakage problems at other test points.

## Figures and Tables

**Table 1:** Distribution of the values of the HW of the S-box output of AES.

HW	0	1	2	3	4	5	6	7	8
Number	1	8	28	56	72	56	28	8	1

**Table 2:** Different Welch’s *t*-test grouping modes.

		Grouping Method
HW	Method I (IHOG, HW Interval)	Byte i HW interval at test positions, i ∈ [0, 15] Subset I HW = {0, 1, 2, 3} Subset II HW = {5, 6, 7, 8}
	Method II [19] (HW value)	Byte i HW value at test positions, i ∈ [0, 15] Subset I HW = j, j ∈ [0, 4] Subset II HW ≠ j, j ∈ [0, 4]
Bit	Method III [12] (Bit value)	Bit i value at test positions, i ∈ [0, 127] Subset I bit i = 0, i ∈ [0, 7] Subset II bit i = 1, i ∈ [0, 7]
Byte	Method IV [19] (Byte value)	Byte i value at test positions, i ∈ [0, 15] Subset I Byte i = j, j ∈ [0, 255] Subset II Byte i ≠ j, j ∈ [0, 255]

**Table 3:** Results on DPA v4.

Byte	Method I (Ours)	Method II [35]	Method III [1]	Method IV [35]
0	**271 **	122	109	91
1	**166**	79	94	84
2	**221**	91	114	52
3	**179**	83	165	73
4	**205**	88	91	82
5	**196**	77	87	47
6	**136**	56	96	66
7	**184**	83	182	64
8	**196**	89	114	44
9	**140**	74	105	87
10	**163**	79	98	56
11	**176**	106	103	75
12	**149**	70	111	67
13	**156**	95	175	45
14	**244**	75	208	65
15	**286**	134	416	71

**Table 4:** Results on AES_HD.

Byte	Method I (Ours)	Method II [35]	Method III [1]	Method IV [35]
0	**42**	11	0	4
1	**59**	12	0	5
2	**50**	12	0	8
3	**71**	26	2	10
4	**19**	5	0	0
5	**39**	21	0	6
6	**57**	14	0	3
7	**37**	13	2	9
8	**18**	12	0	0
9	**82**	28	0	0
10	**45**	12	0	5
11	**59**	9	0	0
12	**53**	9	11	9
13	**35**	12	6	0
14	**53**	20	0	0
15	**69**	13	0	0

**Table 5:** Results on CDS1.

Byte	Method I (Ours)	Method II [35]	Method III [1]	Method IV [35]
0	**309**	146	203	86
1	**431**	204	132	41
2	**340**	155	125	45
3	**441**	246	164	151
4	**216**	129	145	76
5	**523**	248	211	34
6	**427**	195	132	54
7	**262**	109	86	36
8	**43**	30	40	36
9	**568**	276	222	37
10	**419**	193	171	26
11	**440**	213	142	56
12	**36**	24	33	35
13	**326**	95	92	41
14	**449**	196	198	34
15	**486**	297	179	170

**Table 6:** Results on CDS2.

Byte	Method I (Ours)	Method II [35]	Method III [1]	Method IV [35]
0	**70**	34	2	38
1	**48**	22	6	15
2	**59**	36	1	27
3	**50**	26	9	18
4	**49**	27	3	47
5	**80**	36	4	20
6	**55**	33	21	37
7	57	34	**67**	21
8	**65**	30	55	27
9	**55**	22	4	20
10	**73**	41	4	28
11	**51**	22	10	18
12	**49**	19	9	25
13	**63**	31	9	26
14	**57**	23	4	12
15	**53**	29	4	17

**Table 7:** Leakage detection time under different group modes.

The Grouping Mode	The Detection Time per Byte (s)	The Detection Time for 16 Bytes (s)
Method I (ours)	1 × 2 × 900	1 × 2 × 900 × 16
Method II [35]	5 × 2 × 900	5 × 2 × 900 × 16
Method III [1]	8 × 2 × 900	8 × 2 × 900 × 16
Method IV [35]	256 × 2 × 900	256 × 2 × 900 × 16

## Data Availability

The data presented in this study are openly available at https://www.DPAcontest.org/v4/, accessed date 29 March 2026.
